# A thorough *MECP2* mutation analysis

**DOI:** 10.1111/j.1399-0004.2008.01082.x

**Published:** 2008-12

**Authors:** K Ravn, JB Nielsen

**Affiliations:** Kennedy Center, Center for Rett syndromeGlostrup, Denmark

To the Editor:

With great interest we have read the short report of Takahashi et al. entitled, ‘Skewed X chromosome inactivation failed to explain the normal phenotype of a carrier female with *MECP2* mutation resulting in Rett syndrome’.

The report describes a novel missense *MECP2* mutation (P.A447T) detected in a girl with Rett syndrome and her asymptomatic carrier mother. Results from X-chromosome inactivation (XCI) studies performed on DNA from the mother showed a non-random XCI pattern. Further investigation showed that the mother’s predominantly active X-chromosome harbours the mutant allele. The authors speculate how these results could be correlated to the carrier mother’s normal phenotype and her daughter’s Rett phenotype.

We consider that several relevant investigations are missing – especially to justify the title of this report. The authors need to address further genetic aspects before proposing a functional pathogenic nature of A447T.

We lack information regarding the *MECP2* mutation analysis. Was the Rett patient tested according to current knowledge about *MECP2* mutations including testing for mutations in exon 1 or large deletions/duplications in the *MECP2* gene? (1–4) If present, it could explain the Rett syndrome phenotype and then A447T would most likely be a rare variation.

Are there any relatives in this family (siblings to the patient, to her mother or grandparents) who could be tested? Finding A447T in other family members (especially males) with normal phenotype would be a strong indication of a rare variation.

What do protein substitution programs predict? Is A447T predicted to be a tolerated amino acids shift in MeCP2?

It has to be taken into consideration that no other pathogenic missense mutations in the C-terminal of MeCP2 have been published in a Rett patient.
